# Investigation of new candidate genes in retinoblastoma using the TruSight One “clinical exome” gene panel

**DOI:** 10.1002/mgg3.785

**Published:** 2019-06-17

**Authors:** Demet Akdeniz, Seref Bugra Tuncer, Rejin Kebudi, Betul Celik, Gozde Kuru, Seda Kilic, Ozge Sukruoglu Erdogan, Mukaddes Avsar, Sema Buyukkapu Bay, Samuray Tuncer, Hulya Yazici

**Affiliations:** ^1^ Division of Cancer Genetics, Department of Basic Oncology, Oncology Institute Istanbul University Istanbul Turkey; ^2^ Division of Pediatric Hematology‐Oncology, Oncology Institute Istanbul University Istanbul Turkey; ^3^ Division of Pediatric Hematology‐Oncology, Cerrahpasa Medical Faculty Istanbul University‐Cerrahpasa Istanbul Turkey; ^4^ Istanbul Medical Faculty, Department of Ophthalmology Istanbul University Istanbul Turkey

**Keywords:** mutation, next‐generation sequencing, *RB1* gene, retinoblastoma, retinoic acid pathway

## Abstract

**Background:**

Retinoblastoma (Rb) is the most prevalent intraocular pediatric malignancy of the retina. Significant genetic factors are known to have a role in the development of Rb.

**Methods:**

Here, we report the mutation status of 4813 clinically significant genes in six patients with noncarrier of *RB1* gene mutation and having normal *RB1* promoter methylation from three families having higher risk for developing Rb in the study.

**Results:**

A total of 27 variants were detected in the study. Heterozygous missense variants c.1162G > A (p.Gly388Arg) in the *FGFR4* gene; c.559C > T (p.Pro187Ser) in the *NQO1* gene were identified. The family based evaluation of the variants showed that the variant, c.714T > G (p.Tyr238Ter), in the *CLEC7A* gene in first family; the variant, c.55C > T (p.Arg19Ter), in the *APOC3* gene and the variant, c.1171C > T (p.Gln391Ter), in the *MUTYH* gene in second family; and the variant, c.211G > A (p.Gly71Arg), in the *UGT1A1* gene in the third family, were found statistically significant (*p* < 0.05).

**Conclusion:**

This study might be an important report on emphazing the mutational status of other genes in patients without *RB1* gene mutations and having high risk for developing Rb. The study also indicates the interaction between the retinoic acid pathway and Rb oncogenesis for the first time.

## 1 INTRODUCTION

The effects of tumor supressor genes in cancer were first identified in retinoblastoma (Rb) which is a rare pediatric cancer (Knudson, 1971). Therefore, retinoblastoma was described as a model system for the better understanding of the tumor suppressor genes. Rb is the most prevalent intraocular pediatric malignancy of the retina (Jagadeesan, Khetan, & Mallipatna, 2016). Rb is usually reported as two different forms; hereditary in 25%–35%, and nonhereditary in 65%–75%. Eighty‐five per cent of hereditary tumors are detected in the early age (Murphree, Samuel, Harbour, & Mansfield, 2006). Children with bilateral Rb account for approximately 40% of the patients (Draper, Sanders, Brownbill, & Hawkins, 1992). Aproximately, 20% of children diagnosed with bilateral Rb have a family history (Chintagumpala, Chevez‐Barrios, Paysse, Plon, & Hurwitz, 2007). All bilateral tumors are hereditary, some of the unilateral may be hereditary as well. Patients with hereditary Rb have a risk for developing secondary malignancies such as osteosarcoma, soft tissue sarcomas and melanomas (Wong et al., 1997).

The incidence of Rb is higher in developing countries (Pandey, 2014). The cause of this high incidence rate is unknown. Significant genetic factors are known to have a role in the development of Rb. The disease is known to be initiated by the mutations in the retinoblastoma gene (*RB1*) in accordance with the current literature. The *RB1* gene (Gene ID: 5925, OMIM 614041) produces a nuclear protein called pRB weighing 105 kD. This protein functions as a tumor suppressor, and is involved in the cell regulation, proliferation, and prevents rapid or uncontrolled division of cells (Chaussade et al., 2018).

*RB1* gene includes a wide variety of mutations, including single nucleotide variations, small insertions and deletions (INDELs), and large deletions or duplications. The genetic tests include the screening of genome of 27 exons of *RB1* gene, and close intronic areas by Sanger sequencing, and detection of large rearrangements (large deletions, and duplications) by MLPA analysis. However, these methods are time and money consuming. The next‐generation sequencing (NGS) technology is an important research tool which is an effective and high throughput. However, it is unclear how the disease develops in patients who are noncarriers for the mutations of *RB1* gene including large rearrangements after the mutation screening by Sanger method and MLPA analysis. The stuctural alterations of the other genes would be suggested to be responsible in the development of the disease detected in childhood. According to the recent literature, although many gene expression profiles (Chakraborty et al., 2007; Ganguly & Shields, 2010), and methylation levels (Indovina et al., 2010; Livide et al., 2012) were investigated in Rb disease, no information on the structural alterations of different genes were reported. Therefore, the role of the mutations in other genes that may be responsible for the disease occurrence is still unclear in Rb pathogenesis. The aim of this study was to investigate possible candidate genes associated with Rb oncogenesis in retinoblastoma patients without *RB1* gene mutations including INDELS and large rearrangements and having normal *RB1* promoter methylation and having a heavy family history by using NGS‐based technology.

## 2 MATERIALS AND METHODS

### 2.1 Editorial policies and ethical considerations

The study was approved by the Local and Clinical Research Ethics Committee of Istanbul University (Number of ethical approvall: 2016‐360); according to the tenets of the Declaration of Helsinki (JAMA 1997; 277:925‐926). Written informed consent was obtained from all participants or parents of children under 18 years of age. This work was supported by Scientific Research Projects Coordination Unit of Istanbul University (Project number: 21460).

### 2.2 Clinical diagnosis and patients

Five patients diagnosed with Rb and one patient with the retinoma, all diagnosed and treated in the Istanbul University, Oncology Institute, Division of Pediatric Hematology‐Oncology and in the Istanbul University, Istanbul Medical Faculty, Department of Ophtalmology between 2011 and 2016 were enrolled in the study. The blood specimens were collected from the patients without *RB1* mutation including large rearrangements and without *RB1* promoter methylation from three families. In two members of each three families *RB1(*RefSeq NM_000321.2 and chromosome 13 co‐ordinates in hg19) gene mutation was initially screened for small INDEL mutation with Sanger Sequencing and for large rearrengements by MLPA analysis. In the first family; a unilateral Rb patient and his second degree relative with retinoma were tested for *RB1* gene mutation. In the second family; again a unilateral Rb patient, who has fibrosarcoma, and his first degree cousin with retinoblastoma were investigated for *RB1* gene mutation. In the third family; two siblings with bilateral Rb were tested for *RB1* gene mutation. At least two members from the same family who had retinoblastoma or retinoma without *RB1* gene mutations were selected. Thus six patients from three families with two members in each family were selected (Table 1).

**Table 1:** The clinical features of the patients

Family ID	Patient no.^a^	Diagnosis and laterality	Tumor site/stage^b^	Age of diagnosis (months)/gender	Leukocoria	Treatment	OS (year)/final situation	Consanguinity
Fm1	1/II‐7	Unilateral retinoma	L	18/M	−	−	32 years/alive	Uncle
Fm1	1/III‐2	Unilateral Rb	L/Group C	8/M	+	CT; LOT	6 years/alive	Nephew
Fm2	2/IV‐2	Unilateral Rb	L/Group E	8/M	+	CT; RT; Enucleation(L)	11 years/dead due to fibrosarcoma	Cousin
Fm2	2/IV‐7	Bilateral Rb	L/Group A R/Group D	1.5/F	+	CT; LOT	8 years/alive	Cousin
Fm3	3/III‐1	Bilateral Rb	L/Group B R/Group D	7/M	+	CT; LOT; IAC; Enucleation(R)	3 years/alive	Brother
Fm3	3/III‐2	Bilateral Rb	L/Group B R/Group E	7/F	−	CT; LOT; IAC; Enucleation(R)	6 years/alive	Sister

Abbreviations: Fm, family; M, male; F, female; L, left eye; R, right eye; RT, radiotherapy; CT, chemotherapy; IAC, intraarterial chemotherapy; LOT, local ophthalmic treatment; OS, Overall Survival; FS, Final Situation

a Patient numbers are coded according to the order in the family pedigree

b Staging according to IRBC.

### 2.3 DNA sample preparation

The peripheral blood samples were collected from available members of the three families. Genomic DNA of all six patients were searched TruSight One panel of 4813 genes associated with human disease by NGS‐based sequencing technology.

First, lymphocyte isolation was performed from the whole blood samples using the Ficoll (Sigma‐Aldrich, Darmstadt, Germany) separation method. The DNA isolation was performed from the pellets of lymphocytes using the QIAamp DNA mini kit (Qiagen, 40724 Hilden, Germany) in accordance with the manufacturer's instruction. Quantification of genomic DNAs was measured by Qubit fluorimeter (ThermoFisher Scientific, Paisley PA4 9RF, UK) and then the concentration of DNAs was adjusted to 10 ng/µl using 10 mM pH 8.5 Tris‐HCl. The fluorometric measurement was repeated, and the concentration was adjusted to 5 ng/µl with the same buffer solution, and 50 ng was prepared for use.

### 2.4 Library generation and next‐generation sequencing

The TruSight One “clinical exome” panel kit (Illumina, San Diego, CA) was used for sequencing the whole gene regions of 4813 genes associated with human disease in the study. In accordance with the kit protocol; genomic DNA tagmentation, cleaning up of the tagmented DNA, cleaning up of the accumulated DNA, hybridization of the probes, catching the hybridized probes, second hybridization, second catch, cleaning up of the catched library, accumulation of the enriched library, cleaning up of the accumulated enriched library, and bioanalyser device (Agilent, Santa Clara, CA) were performed. The generated library was sequenced on the Illumina NextSeq 500 device (Illumina, San Diego, CA) in accordance with the manufacturer's instructions. The 27 pathogenic variants were identified in selected six patients from three families are indicated position according to reference transcript *ACADS* (NM_000017.3); *APOC3* (NM_000040.2); *ATP6V0A4* (NM_020632.2); *C2* (NM_000063.4); *CFB* (NM_001710.5); *CLEC7A* (NM_197947.2); *CX3CR1* (NM_001171174.1); *DSPP* (NM_014208.3); *FGFR4* (NM_002011.4); *FUT6* (NM_000150.2); *GBE1* (NM_000158.3); *GHRL* (NM_001134944.1); *GNPAT* (NM_014236.3); *HBD* (NM_000519.3); *HFE* (NM_000410.3); *KRT85* (NM_002283.3); *MBL2* (NM_000242.2); *MCCC2* (NM_022132.4); *MUTYH* (NM_001128425.1); *NQO1* (NM_000903.2); *RHAG* (NM_000324.2); *RPGRIP1* (NM_020366.3); *SERPINA1* (NM_001002235.2); *SLC34A1* (NM_003052.4); *TYR* (NM_000372.4); *UGT1A1* (NM_000463.2).

### 2.5 Data analysis and interpretation of the results

The Variant Studio v3.0 (Illumina) software was used for the analysis of data. The data obtained after sequencing from the Illumına NextSeq 500 device were first converted into VCF file format, and the files were uploaded to the software program using the Illumina VariantStudio desktop receiver. The data were annoted in the Illumina VariantStudio program. The comprehensive database of this software catches the explanations in variant, gene, and transcript levels. The variant effect predictor is a central resource for the annotation of the transcript results (McLaren et al., 2016), which is a variant program that uses the databases such as NCBI Reference sequence database (RefSeq) (O'Leary et al., 2016), and the in silico algorithms such as Polymorphism Phenotyping (PolyPhen) (Adzhubei et al., 2010), and SIFT (Kumar, Henikoff, & Ng, 2009). The information about the association with the disease was obtained through the Catalogue of Somatic Mutations in Cancer (COSMIC) (Forbes et al., 2017), from ClinVar database (Landrum et al., 2018), and from the catalogue of the Online Mendelian Inheritance in Man (OMIM) (McKusick, 2007). The resources, dbSNP (Sherry et al., 2001), Exome Aggregation Consortium (ExAC) and Genome Aggregation Database (gnomAD) (Lek et al., 2016), and Ensembl 1000 Genomes Project (Genomes Project et al., 2015) provide information about the occurrence, and frequencies of the variants in a population. The obtained variants were evaluated considering the >Q30 reading quality, and >50 confidence score. All the data about the variants, and information on the algorithms were evaluated in all the related databases. Various filtering options were used for identification of the phenotypes of the variants that were performed annotation procedure. In the study, the variants with particularly pathogenic according to ClinVar records were investigated in details. The variants have not been previously reported in the literature or databases with Rb were identified as candidate variants. The defined variants were labeled in accordance with the recommendation standards of the American College of Medical Genetics, and Genomics (ACMG) (Richards et al., 2008). In order to confirm the all pathogenic variants identified by NGS, PCR amplification, and bidirectional Sanger sequencing was performed using standard reagents and conditions, and oligonucleotide primers flanking the variants.

### 2.6 The analysis of the functional association between genes

The database for annotation, visualization and integrated discovery (DAVID) v.6.8 [Laboratory of Immunopathogenesis and Bioinformatics (LIB)], and STRING Functional Protein Association Network v.10.5 were used for the interpretation of the functional association between the genes that were known to have pathogenic variants after the analysis (Huang da, Sherman, & Lempicki, 2009a, 2009b; Szklarczyk et al., 2017).

### 2.7 Statistical analysis

All clinical and genetic data were evaluated using the IBM Statistical Package for the Social Sciences (SPSS) Statistics v.20 (SPSS Inc., Chicago, IL) program. The Chi‐square test was used to compare the results of VariantStudio analysis for both based on patient, and between the patients for the clinical, and genetic data. The results with a *p* < 0.05 were accepted as statistically significant.

## 3 RESULTS

### 3.1 The clinical and genetic information of the patients

Six patients from three families with two members in each family who were noncarriers of *RB1* gene mutations and normal *RB1* promoter methylation were selected. The cases in the first family consisted of an uncle and a nephew, in the second family consisted of two of five first‐degree cousins, and in the third family consisted of two siblings. The uncle in the first family was diagnosed with unilateral retinoma and has been under follow‐up. Two patients (33.3%) had unilateral Rb, three patients (50%) had bilateral Rb and one (16.7%) had unilateral retinoma. Four patients (66.7%) were male and two were female (33.3%). The median age of the patients was 7.5 months with arange of 1.5–18 months at diagnosis. Four patients (66.7%) had presented with leukocoria and esotropia; one patient (16.7%) had exotropia at diagnosis. One of the patients (2/IV‐2) developed fibrosarcoma as a second malignancy 10^4/12 ^years after the diagnosis of retinoblastoma at the radiation site and died due to progressive disease.

**Figure 1:**
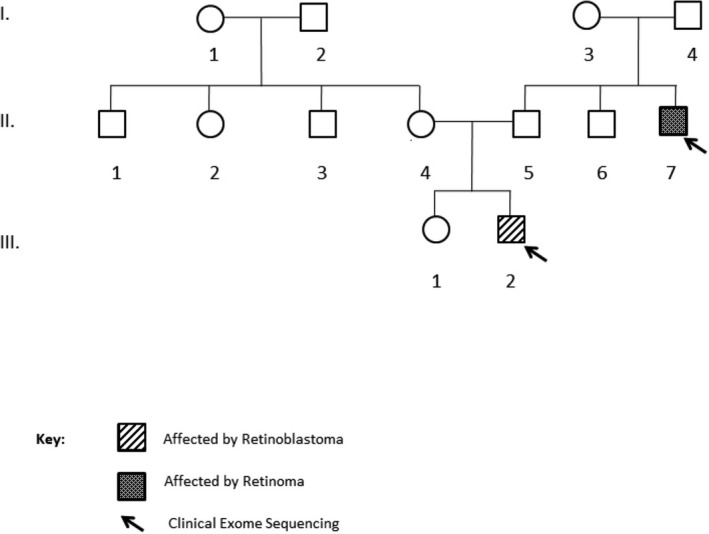
The pedigree of first family. The affected individuals were illustrated with filled box. Two family members marked by arrows were chosen for DNA sequencing, 4813 genes which are clinically important were sequenced by NGS. The sequenced genes were named as a Clinical Exome Sequencing

**Figure 2:**
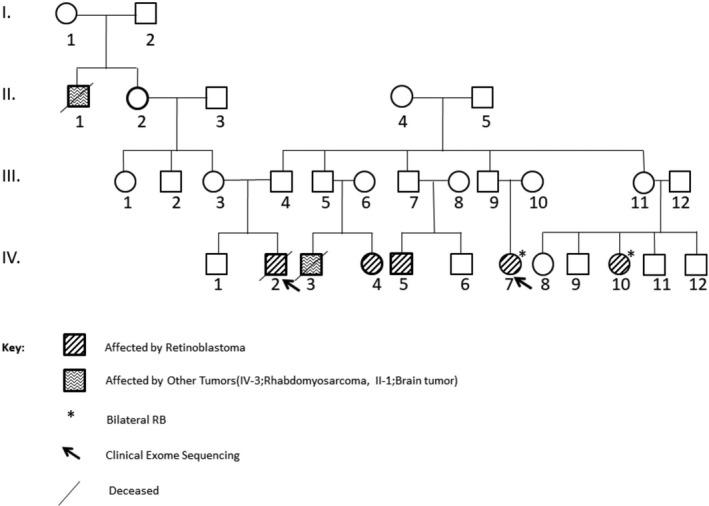
The pedigree of second family. The affected individuals were illustrated with filled box. Two family members marked by arrows were chosen for DNA sequencing, 4813 genes which are clinically important were sequenced by NGS. The sequenced genes were named as a Clinical Exome Sequencing

**Figure 3:**
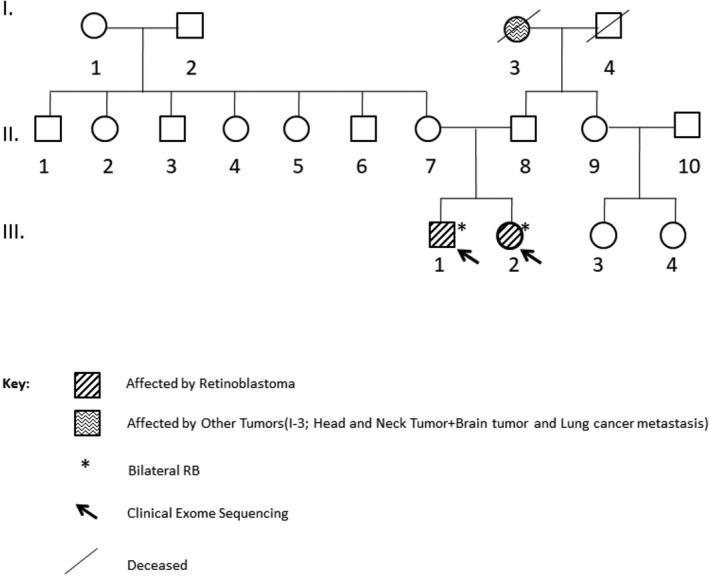
The pedigree of third family. The affected individuals were illustrated with filled box. Two family members marked by arrows were chosen for DNA sequencing, 4813 genes which are clinically important were sequenced by NGS. The sequenced genes were named as a Clinical Exome Sequencing

Four patients, patients 1/III‐2, 2/IV‐7, 3/III‐1, 3/III‐2, recieved systemic chemotherapy (CT) for chemoreduction and local ophthalmic treatment (LOT) (laser, cryotherapy); one patient, 2/IV‐2, had CT and radiotherapy (RT) and underwent enucleation due to relapse. Two patients, 3/III‐1 and 3/III‐2, recieved CT and LOT, on follow‐up developed new lesions, they received intraarterial chemotherapy, due to further progression underwent enucleation. Only one patient, 2/IV‐2, developed fibrosarcoma, 11 years after primary treatment in the irradiated site. The clinical characteristics, treatment and outcome of the six patients are given in Table 1. The pedigrees of families who were included in the study are given in Figures 1, 2, 3.

### 3.2 Evaluation of the analysis results

The mutation status of 4813 clinically significant genes were screened using the TruSight One “clinical exome” panel by NGS in *RB1‐*negative six patients from three families. The number of variants for each patient before, and after the annotation and filtration process are shown in Table 2.

**Table 2:** The numbers of variants found in patients after the process of annotation and filtering

Family ID	Patient no^a^	Number of variants after annotation	Number of variants after filtering	Number of deleterious variants	Number of genes having deleterious variant
Fm1	1/II‐7	121.757	17	15	15
Fm1	1/III‐2	107.327	9	7	7
Fm2	2/IV‐2	60.491	8	8	8
Fm2	2/IV‐7	99.490	13	10	10
Fm3	3/III‐1	108.627	9	5	5
Fm3	3/III‐2	110.976	7	4	4

Abbreviation: Fm, family.

a Patient numbers are coded according to the order in the family pedigree.

A total of 608.668 variants were found in the evaluation of the sequenced data of all patients (1/II‐7, 1/III‐2, 2/IV‐2, 2/IV‐7, 3/III‐1, 3/III‐2). However, the number of these variants decreased to 63 when the variants were filtered according to ClinVar pathogenic records about frameshift, stop gained, stop lost, initiator codon, inframe insertion, inframe deletion, and splice region mutations and according to Polyphen for “damaging” and to SIFT for “deleterious” about missense. Then, 27 pathogenic variants were detected after scanning on ALAMUT, HGMD and dbSNP databases. The information of the variants is shown in Table 3.

**Table 3:** The list of 27 pathogenic mutations

Patient No.	Genes (Reference transcript according to HGVS)	Mutations	dbSNP number	Type of Mutations	Primary Region of Effected in COSMIC	Cited cancer in COSMIC	MAF	SIFT^c^	PolyPhen^d^	ClinVar
1/II‐7; 1/III‐2; 3/III‐2	*ACADS* (NM_000017.3)	c.625G > A (p.Gly209Ser)	rs1799958	missense_variant	Liver; soft tissue; breast	Carcinoma; rhabdomyosarcoma; carcinoma	0.2586	Deleterious (0.01)	Benign (0.342)	Pathogenic: benign
2/IV‐2; 2/IV‐7	*APOC3*^a^ (NM_000040.2)	c.55C > T (p.Arg19Ter)	rs76353203	nonsense_variant	na	na	0.0006032	na	na	Pathogenic
1/II‐7	*ATP6V0A4* (NM_020632.2)	c.1739T > C (p.Met580Thr)	rs3807153	missense_variant	Skin; soft tissue	Malign melanoma; rhabdomyosarcoma	0.06794	Deleterious (0.03)	Benign (0.392)	Pathogenic
1/II‐7	*C2* (NM_000063.4)	c.954G > C (p.Glu318Asp)	rs9332739	missense_variant	Central nervous system; soft tissue	Primitive neuroectodermal tumor‐medulloblastoma; rhabdomyosarcoma	0.03853	Tolerated (0.23)	Probably damaging (0.933)	Pathogenic
1/II‐7	*CFB* (NM_001710.5)	c.26T > A (p.Leu9His)	rs4151667	missense_variant	Soft tissue	Rhabdomyosarcoma	0.03865	Tolerated (score: 0.3)	Probably damaging (0.999)	Pathogenic
1/II‐7; 1/III‐2	*CLEC7A*^a^ (NM_197947.2)	c.714T > G (p.TYR238Ter)	rs16910526	nonsense_variant	Soft tissue	Rhabdomyosarcoma	0.06091	na	na	Pathogenic
1/II‐7; 1/III‐2; 2/IV‐7	*CX3CR1* (NM_001171174.1)	c.935C > T (p.Thr312Met)	rs3732378	missense_variant	Pancreas; soft tissue	Carcinoma; rhabdomyosarcoma	0.1376	Deleterious (0.03)	Benign (0.333)	Pathogenic
2/IV‐2	*DSPP*^b^ (NM_014208.3)	c.202A > T (p.Arg68Trp)	rs36094464	missense_variant	soft tissue	Rhabdomyosarcoma	0.09294	na	Probably damaging (0.992)	Pathogenic
1/II‐7; 2/IV‐2; 2/IV‐7; 3/III‐1; 3/III‐2	*FGFR4* (NM_002011.4)	c.1162G > A (p.Gly388Arg)	rs351855	missense_variant	Thyroid; soft tissue; soft tissue	Other; rhabdomyosarcoma; hemangioblastoma	0.3209	Tolerated (0.2)	Possibly damaging (0.742)	Pathogenic
2/IV‐7	*FUT6* (NM_000150.2)	c.739G > A (p.Glu247Lys)	rs17855739	missense_variant	Soft tissue; hematopoietic and lymphatic tissue	Rhabdomyosarcoma; hematologic tumors	0.08068	Deleterious (0)	Probably damaging (0.917)	Pathogenic
2/IV‐7 3/III‐1; 3/III‐2	*GBE1* (NM_000158.3)	c.986A > G (p.TYR329Cys)	rs80338671	missense_variant	na	na	0.0004343	Deleterious (0)	Probably damaging (0.999)	Pathogenic
2/IV‐7	*GHRL* (NM_001134944.1)	c.178C > A (p.Leu60Met)	rs696217	missense_variant	Soft tissue	Rhabdomyosarcoma	0.08584	Deleterious (score: 0.04)	Probably damaging (1.000)	Pathogenic
1/II‐7	*GNPAT* (NM_014236.3)	c.1556A > G (p.Asp519Gly)	rs11558492	missense_variant	Hematopoietic and lymphatic tissue	Hematologic tumors	0.1608	Deleterious (0.03)	Benign (0.097)	Pathogenic
3/III‐1	*HBD* (NM_000519.3)	c.82G > T (p.Ala28Ser)	rs35152987	missense_variant	na	na	0.002054	Tolerated (0.11)	Possibly damaging (0.68)	Pathogenic
1/II‐7	*HFE* (NM_000410.3)	c.187C > G (p.His63Asp)	rs1799945	missense_variant	Pancreas; soft tissue	Carcinoma;rhabdomyosarcoma	0.1083	Tolerated (0.74)	Probably damaging (0.974)	Pathogenic
1/II‐7; 1/III‐2; 2/IV‐7	*KRT85* (NM_002283.3)	c.233G > A (p.Arg78His)	rs61630004	missense_variant	Thyroid	Other	0.03779	Tolerated (0.38)	Probably damaging (0.991)	Pathogenic
2/IV‐2	*MBL2*^b^ (NM_000242.2)	c.161G > A (p.Gly54Asp)	rs1800450	missense_variant	Skin; soft tissue	Malign melanoma; rhabdomyosarcoma	0.1378	Deleterious (0)	Probably damaging (0.999)	Pathogenic
1/III‐2	*MBL2* (NM_000242.2)	c.154C > T (p.Arg52Cys)	rs5030737	missense_variant	na	na	0.05500	Deleterious (0)	Probably damaging (0.988)	Pathogenic
3/III‐1	*MCCC2* (NM_022132.4)	c.1015G > A (p.Val339Met)	rs150591260	missense_variant	na	na	0.0007506	Deleterious ‐ low confidence (0.01)	Probably damaging (0.952)	Pathogenic
2/IV‐2; 2/IV‐7	*MUTYH*^a^ (NM_001128425.1)	c.1171C > T (p.Gln391Ter)	rs587783057	missense_variant	Colon	Carcinoma	0.00001629	na	na	Pathogenic
1/II‐7; 1/III‐2; 2/IV‐2; 2/IV‐7	*NQO1* (NM_000903.2)	c.559C > T (p.Pro187Ser)	rs1800566	missense_variant	Large_intestine;biliary_tract;prostate;stomach;soft_tissue	Colon; bile tract; prostate; stomach; soft tissue	0.2469	Deleterious (0)	Probably damaging (0.999)	Pathogenic:drug response
1/II‐7	*RHAG* (NM_000324.2)	c.808G > A (p.Val270Ile)	rs16879498	missense_variant	na	na	0.04170	Deleterious (0)	Possibly damaging (0.519)	Pathogenic
1/II‐7; 2/IV‐2	*RPGRIP1* (NM_020366.3)	c.1639G > T (p.Ala547Ser)	rs10151259	missense_variant	na	na	0.2041	Deleterious (0.04)	Benign (0.259)	Pathogenic:benign
1/III‐2	*SERPINA1* (NM_001002235.2)	c.1177C > T (p.Pro393Ser)	rs61761869	missense_variant	na	na	0.0002741	Deleterious (0)	Probably damaging (0.988)	Pathogenic
1/II‐7	*SLC34A1* (NM_003052.4)	c.272_292del21 (p.Val91_Ala97del)	rs199844043	inframe_ deletion	na	na	0	na	na	Pathogenic
1/II‐7; 2/IV‐2; 2/IV‐7	*TYR* (NM_000372.4)	c.1205G > A (p.Arg402Gln)	rs1126809	missense_variant	Skin; esophagus; cervix	Malign melanoma; carcinoma; carcinoma	0.1764	Deleterious (0.03)	Probably damaging (0.941)	Pathogenic
3/III‐1; 3/III‐2	*UGT1A1*^a^ (NM_000463.2)	c.211G > A (p.Gly71Arg)	rs4148323	missense_variant	Soft tissue; hematopoietic and lymphatic tissue	Rhabdomyosarcoma; hematologic tumors	0.02130	Tolerated (score: 0.42)	Probably damaging (0.982)	Pathogenic:likely benign:likely pathogenic:drug response

Abbreviations: COSMIC, The Catalogue of Somatic Mutations in Cancer; MAF; minor allele frequency from the Exome Aggregation Consortium (ExAC) and Genome Aggregation Database (gnomAD) datasets; na, not available.

a Family‐specific pathogenic variants.

b Patient 2/IV‐2 specific pathogenic variants in terms of prognosis and survival.

c SIFT value predication ranges from 0 to 1. Prediction of damaging or tolerated if the score shows ≤ 0.05 or > 0.05, respectively.

d Polyphen value predication ranges from 0 to 1. A variant is appraised qualitatively, as benign (0.00–0.15), possibly damaging (0.16–0.85), or probably damaging (0.86–1.00).

The heterozygous variant in *FGFR4* gene (GRCh37 Chr5:176520243, NM_002011.4:c.1162G > A p.Gly388Arg) commonly detected in five out of six patients (83.3%), was striking. Four patients (66.7%) had a pathogenic variant in *NQO1* gene (GRCh37 Chr16:69745145, NM_000903.2:c.559C > T p.Pro187Ser). Commonly observed variants in three of the six patients (50%) were *ACADS* gene (GRCh37 Chr12:121176083, NM_000017.3:c.625G > A p.Gly209Ser), *CX3CR1* gene (GRCh37 Chr3:39307162, NM_001171174.1:c.935C > T p.Thr312Met), *GBE1* gene (GRCh37 Chr3:81691938, NM_000158.3:c.986A > G p.Tyr329Cys)*, KRT85* gene (GRCh37 Chr12:52760957, NM_002283.3:c.233G > A p.Arg78His), and *TYR* gene (GRCh37 Chr11:89017961, NM_000372.4:c.1205G > A p.Arg402Gln). The presence of mutations in the determined genes in the majority of patients indicates a statistically significant relationship between these genes, and Rb (*p* < 0.05).

The family‐based evaluation of the analysis results showed a variant in *CLEC7A* gene (GRCh37 Chr12:10271087, NM_197947.2:c.714T > G p.Tyr238Ter) in the first family; a variant in *APOC3* gene (GRCh37 Chr11:116701353, NM_000040.2:c.55C > T p.Arg19Ter), and a variant in *MUTYH* gene (GRCh37 Chr1:45797348, NM_001128425.1:c.1171C > T p.Gln391Ter) in the second family, and a variant in *UGT1A1* gene (GRCh37 Chr2:234669144, NM_000463.2:c.211G > A p.Gly71Arg) in the third family were found to be statistically significant (*p* < 0,05). Family‐specific pathogenic variants were shown in Table 3.

The evaluation of the patients in terms of prognosis, and survival showed that the patient 2/IV‐2 was diagnosed with a secondary tumor and died. The comparison of 2/IV‐2, with other patients, showed that there was a pathogenic variant in the *DSPP* gene (GRCh37 Chr4:88533540, NM_014208.3: c.202A > T p.Arg68Trp); and a variant in the *MBL2* gene (GRCh37 Chr10:54531242, NM_000242.2:c.161G > A p.Gly54Asp). Patient 2/IV‐2 specific pathogenic variants in terms of prognosis and survival were shown in Table 3.

### 3.3 The associations of the genes with the metabolic pathways

The data consisting of 27 genes belonging to six patients in the study were uploaded into DAVID, and STRING databases were found associated with the Kyoto Encyclopedia of Genes and Genomes (KEGG), and REACTOME pathways (Table 4).

**Table 4:** The gene sets associated with metabolic pathways

Pathway	Effects of genes on metabolic pathways in cells or org anisms	Associated genes
KEGG Pathway	Complement and coagulation cascade	*C2; SERPINA1; MBL2; CFB*
KEGG Pathway	Fagosome	*ATP6V0A4; CLEC7A; MBL2*
KEGG Pathway	Staphylococcus Aureus Infection	*C2; MBL2; CFB*
REACTOME Pathway	Catalysis	*C2; MBL2*
KEGG Pathway	Tuberculosis	*ATP6V0A4; CLEC7A*
REACTOME Pathway	Regulation of the complement cascade	*C2; CFB*
*KEGG Pathway*	Valine, Leucine, and isoleucine catabolism	*ACADS; MCCC2*
REACTOME Pathway	Spontaneous Separation of the C3 converters	*C2; CFB*
REACTOME Pathway	Catalysis	*C2; CFB*
KEGG Pathway	Starch and sucrose metabolism	*UGT1A1; GBE1*

Abbreviation: KEGG, Kyoto Encyclopedia of Genes and Genomes.

Three particular significant metabolic pathways were detected in DAVID database in the study. Four genes, *C2, CFB, MBL2,* and *SERPINA1* (p: 0.00055) were found effective in complement and coagulation cascade on the immune system, three genes, *C2, CFB, MBL2* (p: 0.008) were found effective in staphylococcus aureus infection and three genes, *ATP6V0A4; CLEC7A, MBL2* (p: 0.05) were found effective in the occurrence of cellular phagocytosis and there was an association between mutations and Rb in the study.

### 3.4 The analysis of protein–protein interactions

**Figure 4:**
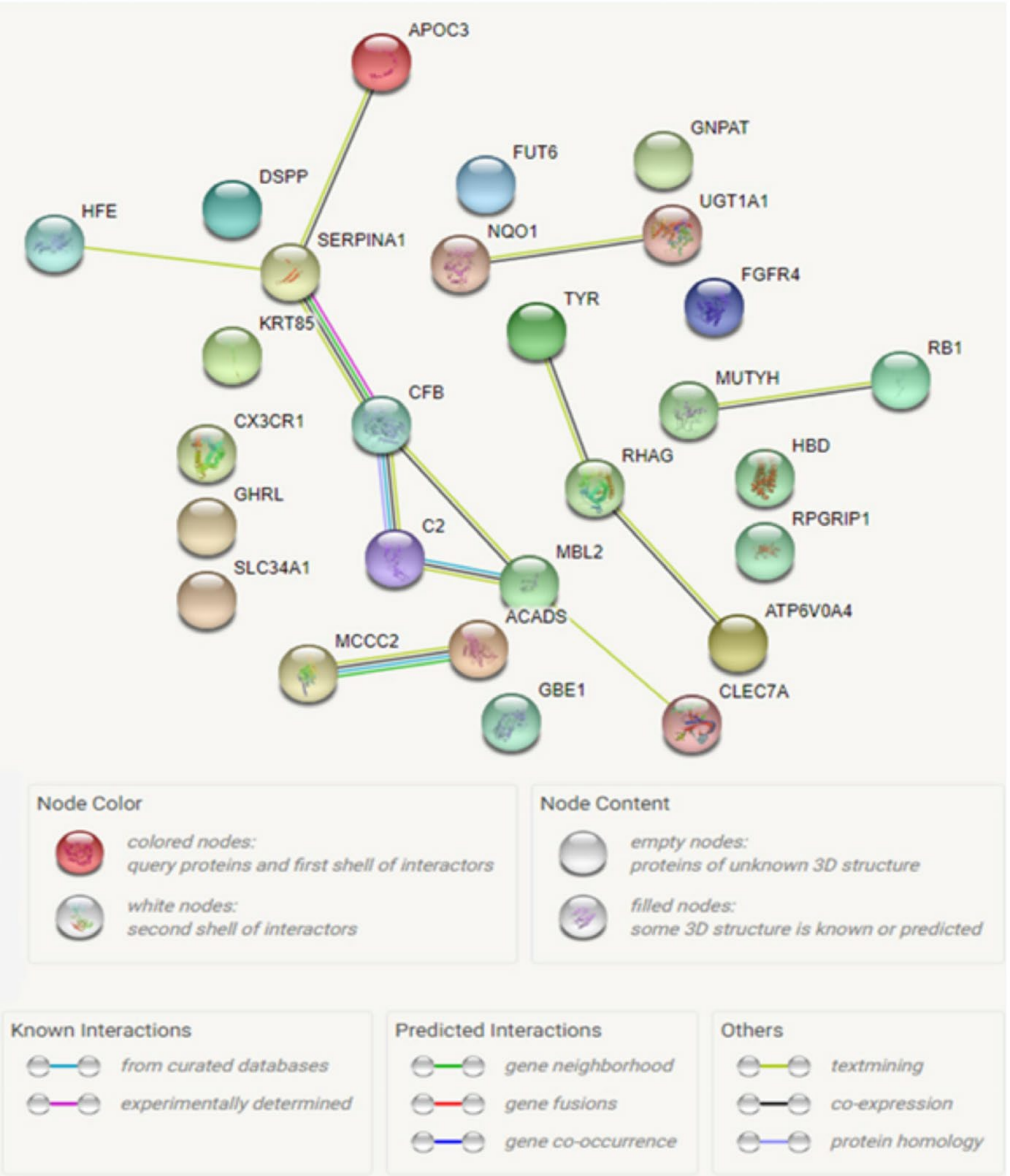
Protein–protein interactions of the genes mutated in the study. Each node represents all proteins produced by a single protein‐encoding gene locus. The edges that link between the proteins, which are determined to be related to each other, showed the protein‐protein interactions

The interactions between proteins in the STRING database were analyzed, and shown in Figure 4. Accordingly, a total of 27 nodes (circles), and 12 edges were identified. The results of the evaluation of this database are intended to be specific and meaningful, that is the proteins contribute to a common function, but this does not mean that they are physically linked to each other. This protein network obtained after the analysis showed a more significant level of protein interactions than expected (p: 0.0000097). This fact means that the protein set obtained in the study has more interactivity than would be expected from a random set of proteins at the same size in the genome. This indicates that the protein group is at least partly biologically involved or associated with each other.

## 4 DISCUSSION

Significant genetic factors are known to have a role in the development of Rb. Development of Rb is thought to be due to mutations in the *RB1* gene. Genetic factors that are responsible for retinoblastoma are not all yet identified in patients who do not have the *RB1* gene mutations. This is an important problem for Rb oncogenesis and need to be investigated. Changes in the number of the copies in the other genes in addition to *RB1* are frequently detected in Rb. An acquisition ranging 4–10 copies in the *MDM4*, *KIF14* (1q32), *MYCN* (2p24), *DEK,* and *E2F3* (6p22) oncogenes, and a loss in the *CDH11* (16q22‐24) tumor supressor gene has been reported (Corson & Gallie, 2007). The different expression profiles of some microRNAs on Rb have been suggested to be related to the let‐7b downregulation (Huang et al., 2007). Single nucleotide deletion, and insertions on the genes *BCOR* and *CREBBP* might be associated with Rb (Kooi et al., 2016). Zhang et al. reported that *SYK* protooncogene was shown to be overexpressed in Rb, and thus may have triggered the development of malignant cell. Furthermore, in the same study, after the full gene sequencing of 11 genes in patients diagnosed with Rb, the mutation merely reported was on the gene *BCOR* (Zhang et al., 2012). According to McEvoy et al., mutations in *BCOR* gene as well chromothripsis as a cause of retinoblastoma (McEvoy et al., 2014). In our study we detected 26 genes that had 27 pathogenic variants that may play a role in the pathogenesis of Rb.

This study differs from other studies in two points. First, none of the six patients had the *RB1* mutation and abnormal *RB1* promoter methylation. Second, all patients had a family history of retinoblastoma since there were two members with Rb in each family all of whom had consanguinity. The results of this study would provide significant clues about the Rb oncogenesis, and could identify new the pathway of Rb disease. The study also indicated the commonly detected genes in patients and the genes specified for the families were found remarkable and informative for Rb disease. Particularly the *CLEC7A* gene in the first family; *APOC3* and *MUTYH* genes in the second family, and *UGT1A1* gene in the third family may be new candidate and specific genes for these families that triggered the occurrence of Rb, since none had a *RB1* gene mutation and abnormal *RB1* promoter methylation. To understand effects of these genes on the heritage of disease based on families and roles in oncogenesis of retinoblastoma, it is recommended to investigate the patients throughout at least three generations in the future.

We detected the c.714T > G (p.Tyr238Ter) variant in the *CLEC7A* gene in the first family (1/II‐7,1/III‐2).* CLEC7A* is also known as the Dectin‐1. According to literature, an association between the *Dectin‐1* immunodeficiency and mucocutaneous fungal infections have been detected in the eye (Klotz, Penn, Negvesky, & Butrus, 2000). Four women from the same family who were immunodeficient were reported to have the c.714T > G (p.Tyr238Ter) mutation in the *CLEC7A* gene and fungal infection (Ferwerda et al., 2009). This mutation was detected in patients 1/II‐7 and 1/III‐2 from the same family in our study. There was no significant history of immunodeficiency or infection in our patients. Moreover, two pathogen recognition receptors, Dectin‐1 and Toll‐like receptor 2 *(TLR2)* metabolizes Vitamin A, and transforms to retinoic acid in dendritic cells (DCs)(Manicassamy et al., 2009). *CLEC7A* gene has been demonstrated to be effective in the retinoic acid pathway. This gene might be a candidate gene in the pathogenesis of the retinoblastoma disease in the first family and also oncogenesis of retinoblastoma.

The pathogenic c.55C > T (p.Arg19Ter) variant was found in *APOC3* gene in the second family (2/IV‐2, 2/IV‐7). *APOC3* is a lipoprotein with a significantly low density. The increase in the level of *APOC3* results in hypertriglyceridemia which is a metabolic complication of the retinoid therapy. Retinoids increase the *APOC3* expression in transcriptional level through retinoid X receptor (RXR). The increase in *APOC3* expression and its release by the retinoids in the liver demonstrating *APOC3* might be a retinoid response gene (Vu‐Dac et al., 1998). The change in this gene which is known to have an association with the retinal pathway was suggested to be associated with Rb. However, pathogenic c.1171C > T (p.Gln391Ter) variant was detected in the *MUTYH* gene in the same family. *MUTYH* is known to have a role in the DNA damage repair. This gene cannot inhibit the accumulation and occurrence of mutation on DNA when it has a mutation. The mutations on the *MUTYH* gene have been associated with the autosomal recessive form of the syndrome of familial adenomatous polyposis (MYH associated polyposis) (Ali et al., 2008). The detection of a pathogenic variant on *MUTYH* gene in two patients, 2/IV‐2 and 2/IV‐7, in our study may suggest a risk for MYH‐associated polyposis, and colon cancer in the future. The patient 2/IV‐2 was diagnosed with unilateral Rb, and died of fibrosarcoma in the proceeding years of life; 2/IV‐7 was diagnosed with bilateral Rb, and three first‐degree cousins in the same family were diagnosed with unilateral Rb, and one cousin was diagnosed with rhabdomyosarcoma; which suggested that this variant might be associated with the Rb disease. The STRING protein‐protein analysis showed that *MUTYH* gene, and *RB1* gene had a significant association. This association between *MUTYH* gene and *RB1* gene may suggest the possibility that this variant might be responsible for the occurrence of Rb in this family. In addition, in families with MUTYH gene mutation exist a risk for a predisposition to juvenile colon cancer as others reported having. To clarify this association, this pathogenic variant must be investigated in future studies in the other individuals diagnosed with Rb in the family and also in large patients cohort and population‐based healthy controls.

The c.211G > A (p.Gly71Arg), pathogenic variant was detected in *UGT1A1* gene in the third family (3/III‐1, and 3/III‐2).* UGT1A1*, performs a chemical reaction named as glucuronidation (Gong et al., 2001). An association was demonstrated on chemical reaction of *UGT1A1* and 13‐cis retinoic acid in the literature. 13‐cis retinoic acid is known as the retinol derivative which organizes numerous biological procedures including embriyogenesis, growth, differentiation, vision, and reproduction (Evans & Kaye, 1999). Twenty‐one functional UGT isoforms, which catalyze the glucuronidation most of which consisting of various environmental carcinogens, nutritional chemopreventives, and anticancer agents in human, have been described (Nagar & Remmel, 2006). The detection of c.211G > A (p.Gly71Arg) pathogenic variant in *UGT1A1* gene in patients 3/III‐1 and 3/III‐2 suggested that this mutation might have triggered the occurrence of cancer by affecting the retinoic acid metabolism in patients.

In addition, the *FGFR4* and *NQO1* genes detected in the majority of the patients might be thought to be effective candidate genes in the Rb etiology and pathogenesis. To understand the exact role of these genes in Rb etiology and pathogenesis, the alterations of these genes must be investigated in large patient groups with the familial segregation and compared with population‐based healthy controls. We detected the c.559C > T (p.Pro187Ser) pathogenic variant in the gene *NQO1* in patients, 1/II‐7, 1/III‐2, 2/IV‐2, and 2/IV‐7. *NQO1* gene is named as the anticancer enzyme because *NQO1* gene protects the cells from oxidative damage. In addition to the protective role in the carcinogenesis, *NQO1* gene functions as the drug metabolizing enzyme in the antitumor treatment. The mutations in this gene were associated with Tardive dyskinesia (TD), an increase in the risk of hematoxocity after exposure to benzene, and predisposition to various cancer types (Smith, 1999; Zai et al., 2010). The modified expression of this protein was detected in various tumors such as lung, bladder, breast, hepatocellular carcinoma, acute myeloid leukemia (AML), colorectal cancer, and gastrointestinal cancers, and in addition it was associated with the Alzheimer's disease (Chao, Zhang, Berthiller, Boffetta, & Hashibe, 2006; Chhetri, King, & Gueven, 2017; Valenzuela et al., 2014). The variant of c.559C > T (p.Pro187Ser) in *NQO1* gene decreased the enzymatic activity and increased the risk of lung cancer. This variant caused the predisposition to bladder and colorectal cancer (Chao et al., 2006). Similarly, c.559C > T (p.Pro187Ser) variant detected in our patients 1/II‐7, 1/III‐2, 2/IV‐2, and 2/IV‐7 was suggested to increase the risk of lung, bladder, and colorectal cancers. The increase in the *NQO1* target gene transcription affected the retinoic acid pathway, and prevent from cancer (Valenzuela et al., 2014). Therefore, the detection of the pathogenic variant of the gene *NQO1* in four patients from two different families suggested that it might be associated with the pathogenesis of Rb. However, c.1162G > A (p.Gly388Arg) variant detected in *FGFR4* gene in our patients 1/II‐7, 2/IV‐2, 2/IV‐7, 3/III‐1, and 3/III‐2. *FGFR4* gene, is a member of fibroblast growth factor (FGF) family which has a role in various mechanisms such as cellular proliferation, differentiation, tissue repair, invasion, regulation of the lipid metabolism, bile acid biosynthesis, glucose intake, Vitamin D metabolism, and phosphate balance. The c.1162G > A (p.Gly388Arg) variant in *FGFR4* gene, and the increase in the *FGFR4* expression were associated with the development of breast, and colon cancer. In addition, it was reported to be statistically associated with the lymph node metastasis, and increased TNM stage, and demonstrated to trigger the cancer progression (Bange et al., 2002). The *FGFR4* expression was associated with pancreatic cancers (Leung, Gullick, & Lemoine, 1994). Cancer progression and tumor cell motility were associated with the c.1162G > A (p.Gly388Arg) change in *FGFR4* gene (Bange et al., 2002). The variant in the gene *FGFR4* was effective in the initiation, and in the progression of prostate cancer (Wang, Stockton, & Ittmann, 2004). *FGFR4* gene is also known with its oncogenic transformation activity which is required in the down‐regulation of the expression of the speed limiting enzyme of CYP7A1 in the synthesis of bile acid as a response to FGF19. Some fibroblast growth factors are known to have neuroprotective effects against the retinal photoreceptor degeneration. The expression of *FGFR4* in the photoreceptors suggested a specific ligand of FGF‐19 might be beneficial. FGF‐19 is important for the development of the ocular tissue, and is a molecule expressed by the embryonic retina. Therefore, the potential role of FGF‐19 has been investigated in many studies in the literature. FGF‐19 had neuroprotective effects on mammalian photoreceptors (Siffroi‐Fernandez, Felder‐Schmittbuhl, Khanna, Swaroop, & Hicks, 2008). Photoreceptor degeneration develops as a pathologic response to numerous environmental and genetic disorders, and causes progressive vision loss and blindness. The hereditary retinal diseases such as retinitis pigmentosa and age associated macular degeneration (AMD) cause significant difficulties in the affected patients. FGF‐19 was expressed by the cells adjacent to photoreceptor layer, and FGF‐19 induced the dose and time‐dependent phosphorylation of *FGFR4* in purified adult photoreceptor cultures, upregulated the expression of the specific transcription factors, and increased the survival (Siffroi‐Fernandez et al., 2008). Therefore, it was suggested to be a beneficial therapeutic approach in the treatment of retinal degeneration. In this regard, our results suggested the c.1162G > A (p.Gly388Arg) pathogenic variant commonly detected in *FGFR4* gene that is known to have a role in cancer progression, and retinal development in patients 1/II‐7, 2/IV‐2, 2/IV‐7, 3/III‐1, and 3/III‐2, might be a candidate mechanism triggering the development of Rb. Furthermore, the common variant was found only in the *FGFR4* gene among the 4813 genes and may be a biomarker of Rb disease. The presence of the gene variants should be investigated with larger patient groups and population‐based healthy controls in the future studies.

In conclusion, in this study we investigated candidate genes that may trigger Rb oncogenesis in six patients with retinoblastoma or retinoma within three families and who did not have a *RB1* gene mutation and abnormal *RB1* promoter methylation. This is the first study suggesting that these genes, *FGFR4, NQO1*, *ACADS CX3CR1*, *GBE1, KRT85,* and *TYR* genes, may play a role in the etiology of Rb. Although, in the literature database these genes were not reported to be involved in Rb promotion, they have found to be associated with the retinoic acid pathway; that has been suggesting to play a role in the Rb oncogenesis. It is recommended that these genes should be investigated in larger cohorts of patients and compared with population‐based healthy controls in the future.

## CONFLICT OF INTEREST

No conflict of interest was declared by the authors.

## References

[mgg3785-bib-0001] Adzhubei, I. A. , Schmidt, S. , Peshkin, L. , Ramensky, V. E. , Gerasimova, A. , Bork, P. , … Sunyaev, S. R. (2010). A method and server for predicting damaging missense mutations. Nature Methods, 7(4), 248–249. 10.1038/nmeth0410-248 20354512 PMC2855889

[mgg3785-bib-0002] Ali, M. , Kim, H. , Cleary, S. , Cupples, C. , Gallinger, S. , & Bristow, R. (2008). Characterization of mutant MUTYH proteins associated with familial colorectal cancer. Gastroenterology, 135(2), 499–507. 10.1053/j.gastro.2008.04.035 18534194 PMC2761659

[mgg3785-bib-0003] Genomes Project, C. , Auton, A. , Brooks, L. D. , Durbin, R. M. , Garrison, E. P. , Kang, H. M. , … Abecasis, G. R. (2015). A global reference for human genetic variation. Nature, 526(7571), 68–74. 10.1038/nature15393 26432245 PMC4750478

[mgg3785-bib-0004] Bange, J. , Prechtl, D. , Cheburkin, Y. , Specht, K. , Harbeck, N. , Schmitt, M. , … Ullrich, A. (2002). Cancer progression and tumor cell motility are associated with the FGFR4 Arg(388) allele. Cancer Research, 62(3), 840–847. 11830541

[mgg3785-bib-0005] Chakraborty, S. , Khare, S. , Dorairaj, S. K. , Prabhakaran, V. C. , Prakash, D. R. , & Kumar, A. (2007). Identification of genes associated with tumorigenesis of retinoblastoma by microarray analysis. Genomics, 90(3), 344–353. 10.1016/j.ygeno.2007.05.002 17604597

[mgg3785-bib-0006] Chao, C. , Zhang, Z. F. , Berthiller, J. , Boffetta, P. , & Hashibe, M. (2006). NAD(P)H:Quinone oxidoreductase 1 (NQO1) Pro187Ser polymorphism and the risk of lung, bladder, and colorectal cancers: A meta‐analysis. Cancer Epidemiology, Biomarkers & Prevention, 15(5), 979–987. 10.1158/1055-9965.EPI-05-0899 16702380

[mgg3785-bib-0007] Chaussade, A. , Millot, G. , Wells, C. , Brisse, H. , Lae, M. , Savignoni, A. , … Houdayer, C. (2018). Correlation between *RB1*germline mutations and second primary malignancies in hereditary retinoblastoma patients treated with external beam radiotherapy. European Journal of Medical Genetics, 62(3), 217 10.1016/j.ejmg.2018.07.017 30031154

[mgg3785-bib-0008] Chhetri, J. , King, A. E. , & Gueven, N. (2017). Alzheimer's disease and NQO1: Is there a link? Current Alzheimer Research, 10.2174/1567205014666170203095802 28164770

[mgg3785-bib-0009] Chintagumpala, M. , Chevez‐Barrios, P. , Paysse, E. A. , Plon, S. E. , & Hurwitz, R. (2007). Retinoblastoma: Review of current management. The Oncologist, 12(10), 1237–1246. 10.1634/theoncologist.12-10-1237 17962617

[mgg3785-bib-0010] Corson, T. W. , & Gallie, B. L. (2007). One hit, two hits, three hits, more? Genomic changes in the development of retinoblastoma. Genes, Chromosomes & Cancer, 46(7), 617–634. 10.1002/gcc.20457 17437278

[mgg3785-bib-0011] da Huang, W. , Sherman, B. T. , & Lempicki, R. A. (2009a). Bioinformatics enrichment tools: Paths toward the comprehensive functional analysis of large gene lists. Nucleic Acids Research, 37(1), 1–13. 10.1093/nar/gkn923 19033363 PMC2615629

[mgg3785-bib-0012] da Huang, W. , Sherman, B. T. , & Lempicki, R. A. (2009b). Systematic and integrative analysis of large gene lists using DAVID bioinformatics resources. Nature Protocols, 4(1), 44–57. 10.1038/nprot.2008.211 19131956

[mgg3785-bib-0013] Draper, G. J. , Sanders, B. M. , Brownbill, P. A. , & Hawkins, M. M. (1992). Patterns of risk of hereditary retinoblastoma and applications to genetic counselling. British Journal of Cancer, 66(1), 211–219. 10.1038/bjc.1992.244 1637670 PMC1977909

[mgg3785-bib-0014] Evans, T. R. , & Kaye, S. B. (1999). Retinoids: Present role and future potential. British Journal of Cancer, 80(1–2), 1–8. 10.1038/sj.bjc.6690312 PMC2362988 10389969

[mgg3785-bib-0015] Ferwerda, B. , Ferwerda, G. , Plantinga, T. S. , Willment, J. A. , van Spriel, A. B. , Venselaar, H. , … Netea, M. G. (2009). Human dectin‐1 deficiency and mucocutaneous fungal infections. New England Journal of Medicine, 361(18), 1760–1767. 10.1056/NEJMoa0901053 19864674 PMC2773015

[mgg3785-bib-0016] Forbes, S. A. , Beare, D. , Boutselakis, H. , Bamford, S. , Bindal, N. , Tate, J. , … Campbell, P. J. (2017). COSMIC: Somatic cancer genetics at high‐resolution. Nucleic Acids Research, 45(D1), D777–D783. 10.1093/nar/gkw1121 27899578 PMC5210583

[mgg3785-bib-0017] Ganguly, A. , & Shields, C. L. (2010). Differential gene expression profile of retinoblastoma compared to normal retina. Molecular Vision, 16, 1292–1303. 20664703 PMC2904042

[mgg3785-bib-0018] Gong, Q. H. , Cho, J. W. , Huang, T. , Potter, C. , Gholami, N. , Basu, N. K. , … Popescu, N. C. (2001). Thirteen UDPglucuronosyltransferase genes are encoded at the human UGT1 gene complex locus. Pharmacogenetics, 11(4), 357–368. 10.1097/00008571-200106000-00011 11434514

[mgg3785-bib-0019] Huang, J. C. , Babak, T. , Corson, T. W. , Chua, G. , Khan, S. , Gallie, B. L. , … Morris, Q. D. (2007). Using expression profiling data to identify human microRNA targets. Nature Methods, 4(12), 1045–1049. 10.1038/nmeth1130 18026111

[mgg3785-bib-0020] Indovina, P. , Acquaviva, A. , De Falco, G. , Rizzo, V. , Onnis, A. , Luzzi, A. , … Giordano, A. (2010). Downregulation and aberrant promoter methylation of p16INK4A: A possible novel heritable susceptibility marker to retinoblastoma. Journal of Cellular Physiology, 223(1), 143–150. 10.1002/jcp.22019 20039270

[mgg3785-bib-0021] Jagadeesan, M. , Khetan, V. , & Mallipatna, A. (2016). Genetic perspective of retinoblastoma: From present to future. Indian Journal of Ophthalmology, 64(5), 332–336. 10.4103/0301-4738.185585 27380971 PMC4966369

[mgg3785-bib-0022] Klotz, S. A. , Penn, C. C. , Negvesky, G. J. , & Butrus, S. I. (2000). Fungal and parasitic infections of the eye. Clinical Microbiology Reviews, 13(4), 662–685. 10.1128/CMR.13.4.662 11023963 PMC88956

[mgg3785-bib-0023] Knudson, A. G. Jr (1971). Mutation and cancer: Statistical study of retinoblastoma. Proceedings of the National Academy of Sciences of the USA, 68(4), 820–823. 10.1073/pnas.68.4.820 5279523 PMC389051

[mgg3785-bib-0024] Kooi, I. E. , Mol, B. M. , Massink, M. P. , Ameziane, N. , Meijers‐Heijboer, H. , Dommering, C. J. , … Dorsman, J. C. (2016). Somatic genomic alterations in retinoblastoma beyond *RB1* are rare and limited to copy number changes. Scientific Reports, 6, 25264 10.1038/srep25264 27126562 PMC4850475

[mgg3785-bib-0025] Kumar, P. , Henikoff, S. , & Ng, P. C. (2009). Predicting the effects of coding non‐synonymous variants on protein function using the SIFT algorithm. Nature Protocols, 4(7), 1073–1081. 10.1038/nprot.2009.86 19561590

[mgg3785-bib-0026] Landrum, M. J. , Lee, J. M. , Benson, M. , Brown, G. R. , Chao, C. , Chitipiralla, S. , … Maglott, D. R. (2018). ClinVar: Improving access to variant interpretations and supporting evidence. Nucleic Acids Research, 46(D1), D1062–D1067. 10.1093/nar/gkx1153 29165669 PMC5753237

[mgg3785-bib-0027] Lek, M. , Karczewski, K. J. , Minikel, E. V. , Samocha, K. E. , Banks, E. , Fennell, T. , … Exome Aggregation, C. (2016). Analysis of protein‐coding genetic variation in 60,706 humans. Nature, 536(7616), 285–291. 10.1038/nature19057 27535533 PMC5018207

[mgg3785-bib-0028] Leung, H. Y. , Gullick, W. J. , & Lemoine, N. R. (1994). Expression and functional activity of fibroblast growth factors and their receptors in human pancreatic cancer. International Journal of Cancer, 59(5), 667–675. 10.1002/ijc.2910590515 7960240

[mgg3785-bib-0029] Livide, G. , Epistolato, M. C. , Amenduni, M. , Disciglio, V. , Marozza, A. , Mencarelli, M. A. , … Ariani, F. (2012). Epigenetic and copy number variation analysis in retinoblastoma by MS‐MLPA. Pathology & Oncology Research, 18(3), 703–712. 10.1007/s12253-012-9498-8 22278416

[mgg3785-bib-0030] Manicassamy, S. , Ravindran, R. , Deng, J. , Oluoch, H. , Denning, T. L. , Kasturi, S. P. , … Pulendran, B. (2009). Toll‐like receptor 2‐dependent induction of vitamin A‐metabolizing enzymes in dendritic cells promotes T regulatory responses and inhibits autoimmunity. Nature Medicine, 15(4), 401–409. 10.1038/nm.1925 PMC2768543 19252500

[mgg3785-bib-0031] McEvoy, J. , Nagahawatte, P. , Finkelstein, D. , Richards‐Yutz, J. , Valentine, M. , Ma, J. , … Dyer, M. A. (2014). *RB1* gene inactivation by chromothripsis in human retinoblastoma. Oncotarget, 5(2), 438–450. 10.18632/oncotarget.1686 24509483 PMC3964219

[mgg3785-bib-0032] McKusick, V. A. (2007). Mendelian Inheritance in Man and its online version, OMIM. The American Journal of Human Genetics, 80(4), 588–604. 10.1086/514346 17357067 PMC1852721

[mgg3785-bib-0033] McLaren, W. , Gil, L. , Hunt, S. E. , Riat, H. S. , Ritchie, G. R. , Thormann, A. , … Cunningham, F. (2016). The ensembl variant effect predictor. Genome Biology, 17(1), 122 10.1186/s13059-016-0974-4 27268795 PMC4893825

[mgg3785-bib-0034] Murphree, A. L. , Samuel, M. , Harbour, J. W. , & Mansfield, N. C. (2006). Retinoblastoma (4th ed.). Philadelphia, PA: Mosby Elsevier.

[mgg3785-bib-0035] Nagar, S. , & Remmel, R. P. (2006). Uridine diphosphoglucuronosyltransferase pharmacogenetics and cancer. Oncogene, 25(11), 1659–1672. 10.1038/sj.onc.1209375 16550166

[mgg3785-bib-0036] O'Leary, N. A. , Wright, M. W. , Brister, J. R. , Ciufo, S. , Haddad, D. , McVeigh, R. , … Pruitt, K. D. (2016). Reference sequence (RefSeq) database at NCBI: Current status, taxonomic expansion, and functional annotation. Nucleic Acids Research, 44(D1), D733–745. 10.1093/nar/gkv1189 26553804 PMC4702849

[mgg3785-bib-0037] Pandey, A. N. (2014). Retinoblastoma: An overview. Saudi Journal of Ophthalmology, 28(4), 310–315. 10.1016/j.sjopt.2013.11.001 25473349 PMC4250503

[mgg3785-bib-0038] Richards, C. S. , Bale, S. , Bellissimo, D. B. , Das, S. , Grody, W. W. , Hegde, M. R. , …Molecular Subcommittee of the ACMG Laboratory Quality Assurance Committee. (2008). ACMG recommendations for standards for interpretation and reporting of sequence variations: Revisions 2007. Genetics in Medicine, 10(4), 294–300. 10.1097/GIM.0b013e31816b5cae 18414213

[mgg3785-bib-0039] Sherry, S. T. , Ward, M. H. , Kholodov, M. , Baker, J. , Phan, L. , Smigielski, E. M. , & Sirotkin, K. (2001). dbSNP: The NCBI database of genetic variation. Nucleic Acids Research, 29(1), 308–311. 10.1093/nar/29.1.308 11125122 PMC29783

[mgg3785-bib-0040] Siffroi‐Fernandez, S. , Felder‐Schmittbuhl, M. P. , Khanna, H. , Swaroop, A. , & Hicks, D. (2008). FGF19 exhibits neuroprotective effects on adult mammalian photoreceptors in vitro. Investigative Ophthalmology & Visual Science, 49(4), 1696–1704. 10.1167/iovs.07-1272 18385093

[mgg3785-bib-0041] Smith, M. T. (1999). Benzene, NQO1, and genetic susceptibility to cancer. Proceedings of the National Academy of Sciences of the USA, 96(14), 7624–7626. 10.1073/pnas.96.14.7624 10393869 PMC33590

[mgg3785-bib-0042] Szklarczyk, D. , Morris, J. H. , Cook, H. , Kuhn, M. , Wyder, S. , Simonovic, M. , … von Mering, C. (2017). The STRING database in 2017: Quality‐controlled protein‐protein association networks, made broadly accessible. Nucleic Acids Research, 45(D1), D362–D368. 10.1093/nar/gkw937 27924014 PMC5210637

[mgg3785-bib-0043] Valenzuela, M. , Glorieux, C. , Stockis, J. , Sid, B. , Sandoval, J. M. , Felipe, K. B. , … Buc Calderon, P. (2014). Retinoic acid synergizes ATO‐mediated cytotoxicity by precluding Nrf2 activity in AML cells. British Journal of Cancer, 111(5), 874–882. 10.1038/bjc.2014.380 25003661 PMC4150280

[mgg3785-bib-0044] Vu‐Dac, N. , Gervois, P. , Torra, I. P. , Fruchart, J. C. , Kosykh, V. , Kooistra, T. , … Staels, B. (1998). Retinoids increase human apo C‐III expression at the transcriptional level via the retinoid X receptor. Contribution to the hypertriglyceridemic action of retinoids. Journal of Clinical Investigation, 102(3), 625–632. 10.1172/JCI1581 9691099 PMC508923

[mgg3785-bib-0045] Wang, J. , Stockton, D. W. , & Ittmann, M. (2004). The fibroblast growth factor receptor‐4 Arg388 allele is associated with prostate cancer initiation and progression. Clinical Cancer Research, 10(18 Pt 1), 6169–6178. 10.1158/1078-0432.CCR-04-0408 15448004

[mgg3785-bib-0046] Wong, F. L. , Boice, J. D. Jr , Abramson, D. H. , Tarone, R. E. , Kleinerman, R. A. , Stovall, M. , … Li, F. P. (1997). Cancer incidence after retinoblastoma. Radiation dose and sarcoma risk. JAMA, 278(15), 1262–1267. 10.1001/jama.1997.03550150066037 9333268

[mgg3785-bib-0047] Zai, C. C. , Tiwari, A. K. , Basile, V. , de Luca, V. , Muller, D. J. , Voineskos, A. N. , … Kennedy, J. L. (2010). Oxidative stress in tardive dyskinesia: Genetic association study and meta‐analysis of NADPH quinine oxidoreductase 1 (NQO1) and Superoxide dismutase 2 (SOD2, MnSOD) genes. Progress in Neuro‐Psychopharmacology and Biological Psychiatry, 34(1), 50–56. 10.1016/j.pnpbp.2009.09.020 19778569

[mgg3785-bib-0048] Zhang, J. , Benavente, C. A. , McEvoy, J. , Flores‐Otero, J. , Ding, L. , Chen, X. , … Dyer, M. A. (2012). A novel retinoblastoma therapy from genomic and epigenetic analyses. Nature, 481(7381), 329–334. 10.1038/nature10733 22237022 PMC3289956

